# Beyond Invasion: How *Phragmites australis* Modifies Soil Architecture and Carbon Storage in Long Island Sound Salt Marshes

**DOI:** 10.3390/biology15040315

**Published:** 2026-02-11

**Authors:** Sharon N. Kahara, Precious F. Attah, Ritwik Negi

**Affiliations:** Department of Biology and Environmental Science, University of New Haven, West Haven, CT 06516, USArnegi@newhaven.edu (R.N.)

**Keywords:** blue carbon, bulk density, coastal resilience, Connecticut, *Phragmites australis*, *Spartina alterniflora*, *Sporobolus alterniflorus*, total organic carbon

## Abstract

Coastal salt marshes are essential for climate change mitigation due to their high carbon storage capacity, which is influenced by soil type, hydrology, and floristic composition. Over the past century, invasive *Phragmites australis* has displaced native *Sporobolus alterniflorus* (formerly *Spartina alterniflora*) across salt marshes on the Long Island Sound, and it is widely hypothesized that its larger biomass and rapid growth enhance soil carbon sequestration. This study tested that hypothesis by comparing total organic carbon stocks and physical soil properties in two southern Connecticut marshes over multiple seasons. Our results show that mean soil bulk density was significantly higher under *P. australis* than *S. alterniflorus* at both locations. However, this did not translate to superior carbon storage. Analysis showed a significant seasonal effect but no significant overall difference in median TOC between species, indicating that *P. australis* is competitive in total mass only due to its higher soil density. Notably, Levene’s test for homogeneity of variance was significant (*p* = 0.039), revealing that *P. australis* creates highly heterogeneous “hot spots” of carbon storage compared to the relatively uniform distribution found in native stands. These findings suggest that while *P. australis* invasion results in a more physically dense and potentially resilient marsh platform—relevant for surviving sea-level rise and filtering nutrient runoff—it may simultaneously compromise the stability and uniformity of regional carbon sinks. Management strategies should consider these tradeoffs when prioritizing the protection of native *S. alterniflorus* for consistent carbon sequestration.

## Simple Summary

This study investigated soil total organic carbon stocks and physical properties under invasive *Phragmites australis* and native *Sporobolus alterniflorus* (formerly *Spartina alterniflora*) in southern Connecticut tidal marshes. The findings challenge the assumption that *P. australis* invasion necessarily increases carbon sequestration. While *P. australis* soils consistently exhibited significantly higher and more variable bulk density and dry weight, this did not result in a consistent carbon storage advantage. Notably, *P. australis* created highly heterogeneous localized concentrations of carbon compared to the more consistent distribution in native stands. We conclude that while *P. australis* provides a denser soil structure that may increase resilience against sea-level rise, its complex impact on carbon dynamics may reduce the reliability of these marshes as consistent blue carbon sinks. Management strategies must weigh the benefit of geomorphic stability against the loss of uniform carbon sequestration and native biodiversity.

## 1. Introduction

Coastal salt marshes are among the most productive and valuable ecosystems globally, providing essential services such as critical habitats for commercially important fisheries, storm surge buffers and carbon sequestration [1]. Like other blue carbon systems, salt marshes’ ability to capture and store carbon is directly related to their proximity to the marine ecosystems and hydrology. Despite their ecological significance, salt marshes of the northeastern United States are facing escalating threats that challenge both their functional integrity and physical persistence. Foremost among these are accelerating sea-level rise (SLR), which threatens to drown marshes that cannot accrete vertically [2]; shoreline encroachment [3]; and excess nutrient loading, which can compromise the root structure necessary to hold marsh soil together [4].

Compounding these global change stressors is the widespread proliferation of the invasive common reed, *Phragmites australis* (Cav.) Trin. ex Steud. subsp. *australis* (hereafter *P. australis*). While the native subspecies, *P. australis* (Cav.) Trin. ex Steud. subsp. *americanus Saltonst.*, P.M. Peterson and Soreng, has existed in North American wetlands for millennia [5], the non-native invasive lineage—introduced from Europe by the late-1800s [6]—is recognized as one of the most problematic invasive wetland plants in eastern North America [7]. While native and invasive *P. australis* can coexist, they exhibit distinct physiological differences; invasive Phragmites produce larger inflorescences, leaves, and heights [8,9]. Crucially, this invasion represents more than a biological displacement of native flora; it drives a fundamental restructuring of marsh geomorphology and soil architecture.

Invasive *P. australis* forms dense, monotypic stands with high above-ground biomass that significantly alter local hydrology by trapping mineral sediment and rapidly elevating the marsh surface [10]. While *P. australis* is typically associated with higher elevations to avoid high salinity, it can spread clonally into the low marsh [11,12], bringing its robust root architecture into zones previously dominated by *Sporobolus alterniflorus* (Loisel.) P.M. Peterson and Saarela (formerly *Spartina alterniflora*). This structural modification is critical in the context of erosion; while nutrient enrichment has been shown to weaken the peat fabric of *Sporobolus alterniflorus* (hereafter *S. alterniflorus*) marshes leading to creek bank collapse [4], *P. australis* may offer a different geomorphic trajectory. Understanding how the physical density and stability of the soil matrix change during this invasion is paramount to effective marsh management, particularly as physical resilience to wave energy becomes as valuable as biological diversity.

Despite its reputation as a biological threat, invasive *P. australis* has been widely reported to provide superior ecosystem benefits relative to indigenous counterparts like *S. alterniflorus* and *Sporobolus pumilus* (Roth) P.M. Peterson and Saarela. (formerly *Spartina patens* (Aiton) Muhl), specifically regarding sediment stabilization and accretion [13,14]. A study conducted at a St. Lawrence Estuary marsh found that invasive *P. australis* contributed more to soil volume and carbon stock than native Spartina spp. [15]. Similarly, ref. [16] found that *P. australis* produced far greater above- and below-ground biomass than *S. patens*. Coupled with slower decomposition rates, it was hypothesized that this robust biomass production would lead to increased carbon sequestration and a physically denser peat layer. However, recent research suggests that anthropogenically driven changes, such as nutrient enrichment, may complicate this picture, potentially resulting in soil organic carbon (SOC) loss [17,18,19].

Therefore, evaluating the trade-offs of this invasion requires looking beyond simple carbon quantities to the stability of the soil itself. While carbon sequestration rates are often reported as offsetting greenhouse gas emissions, carbon stocks provide a clearer indication of the ecosystem’s potential to mitigate climate change [20]. Furthermore, the physical properties of these stocks—specifically bulk density—determine the marsh’s ability to resist erosion. In this study, we compared total organic carbon (TOC) stocks and physical soil properties in locations dominated by *P. australis* and *S. alterniflorus* at a salt marsh in southern Connecticut, aiming to determine if long term shifts in vegetation species composition alters the soil architecture required to withstand rising seas.

The overall objective of this study was to evaluate the functional shifts caused by encroachment by invasive *Phragmites australis* over *Sporobolus alterniflorus* in Long Island Sound salt marshes. Primarily, the research sought to determine whether the higher biomass and rapid growth of Phragmites significantly enhance TOC sequestration, thereby altering the soil’s biochemical stoichiometry. Additionally, the study quantified soil physical characteristics to determine how these changes may have influenced the marsh’s structural integrity.

## 2. Materials and Methods

### 2.1. Study Area

**Figure 1:**
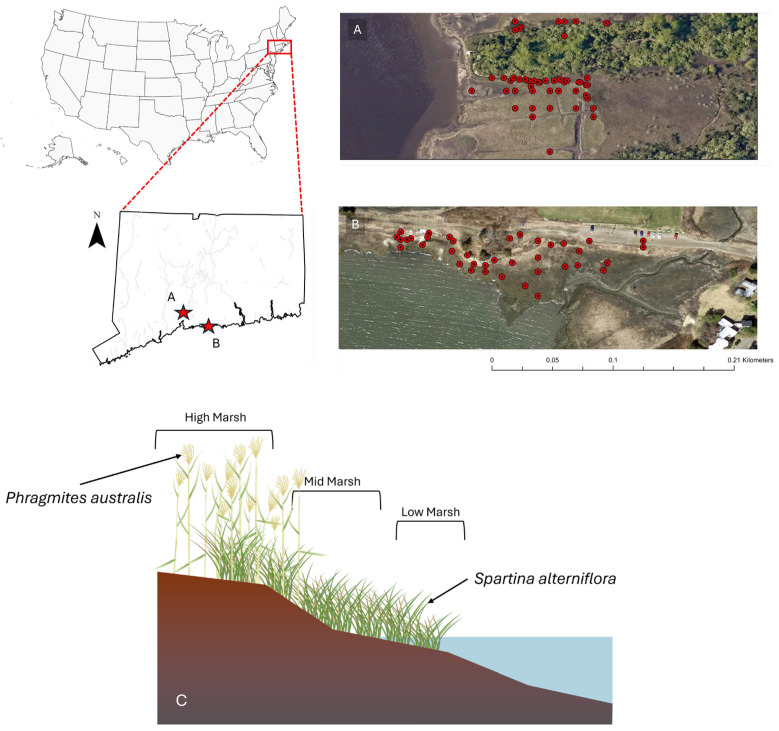
Soil sampling locations at (**A**) Quinnipiac Meadows and (**B**) Branford marsh. Distribution of dominant vegetation in a typical tidal salt marsh profile in southern Connecticut (**C**).

Soil samples were collected at two tidal salt marshes (Figure 1) located in southern Connecticut; namely, Branford Trolley Trail (41°16′9.28″ N, 72°45′18.60″ W) and Quinnipiac Meadows (Eugene B. Fargeorge) Preserve located in the lower section of the Quinnipiac River (41°19′00″ N, 72°52′50″ W). Both marshes exchange tides with the Long Island Sound and share a similar temperate climate with an average annual temperature of 11.3 °C, and average annual rainfall of 1273 mm. Temperatures peak in July, with highs of 23.2 °C, while the lowest average temperatures occur in January, at around −0.5 °C. The lowest amount of rainfall occurs in July with an average of 81 mm, while the greatest amount of precipitation occurs in December, with an average of 130 mm [21]. The region experiences four distinct seasons with cold winters (−7 to 4 °C) with occasional snow and ice, though tidal flow prevents full soil freezing. Spring (4 to 18 °C) brings increased rainfall and promotes plant growth. Summers (18 to 29 °C) are warm and humid, with peak marsh productivity and frequent thunderstorms. Fall (10 to 21 °C) introduces potential for early frost and coastal storm surges, affecting both vegetation and soil chemistry [22,23]. At the time of this study, Branford had an average tidal range of about 1.5 m [23], whereas Quinnipiac Meadows experiences a much greater tidal range of about 2.8 m [24,25]. Marsh vegetation at Branford is dominated by stands of *P. australis*, *S. alterniflorus* and *S. patens* interspersed with smaller patches of *Distichlis spicata. P. australis* stands occupy higher elevations of the marsh and distinctively along recently disturbed areas such as gravel pathways, roads and railroad tracks. The gravel path along the northern perimeter of the study site is frequented by human traffic and often littered with animal feces. The elevation of the gravel path is approximately 0.7 m above sea level (m.a.s.l) descending to sea level at the lowest southern boundary. At Quinnipiac Meadows, the marsh contains *S. alterniflorus* and *P. australis* backed by coastal forest. Branford marsh soils were dominated by Westbrook mucky peat and Quiambog silt loam, intertidal while Quinnipiac Meadows soils were mainly Westbrook mucky peat in the high- and mid-marsh areas and Pishagqua silt loam in the low marsh [26]. Invasive *P. australis* was identified in the field by examining the height of the plant and size of inflorescence which is longer and denser than native species [27].

### 2.2. Sample Collection

We employed a stratified random sampling approach to collect soil cores at both sites. Soil cores were stratified and equally distributed by plant type in zones dominated by *P. australis* and *S. alterniflorus*, respectively. In some instances, *P. australis* or *S. alterniflorus* soils were collected under plants growing in transition zones where both species were present, but we were careful to core immediately adjacent to or under plants of one species. At Branford, samples also captured a range of elevations with equal distribution across each of three zones (Figure 1). Determination of marsh zone boundaries was based on a perpendicular transect originating from the highest elevation (areas that remain dry even at peak high tide) to the water’s edge. Vegetation composition was a secondary determent of marsh zones based on plant inundation and salinity tolerances [27]. High marsh zones lay adjacent to a walking path (approximately 0.7 m.a.s.l), to the lowest areas as close as possible to the water’s edge (0 m.a.s.l) to assess the influence of inundation depth, flood frequency and human disturbance on carbon stocks. Sample locations at the lowest elevations by the water’s edge experienced deeper flooding, more frequent flooding and less disturbance than the sites closer to the walking path at the northern border. A 5.5 cm diameter stainless steel regular soil auger was used to collect soil samples with minimum compaction to a depth of 30 cm. Surface dead vegetative matter such as leaves, rhizomes and roots were removed from the sample in the field. Soil cores were stored in polyethylene Ziploc^®^ bags (S. C. Johnson & Son, Inc., Racine, WI, USA) and stored as in the previous mentioned methods.

In 2024, samples were collected from Quinnipiac Meadows on three occasions over three seasons (spring, summer and fall) in 2024, with 20 samples collected at each visit with an equal number of samples collected under each species. Soil samples were carefully collected to minimize compaction from the rhizosphere. Due to drier conditions in 2024 and to avoid damaging soil structure, the collection depths varied between 20–30 cm using a 2.5 cm diameter stainless-steel soil corer [28].

### 2.3. Sample Processing and Analysis

In both Branford and Quinnipiac Meadows, dead vegetative matter including leaves and rhizomes were removed from the surface of the soils prior to coring. Samples were transported in a cooler containing dry ice to maintain a temperature of approximately 4 °C in the field upon return to the University of New Haven where they were immediately stored at −20 °C until processing. During processing, entire soil samples from each site were thawed, homogenized and weighed in grams to obtain wet weight. Samples were then dried at 60 °C for 24 h and re-weighed to obtain dry weight. For all cores, dry bulk density was calculated by dividing the dry weight by the initial volume. Initial volume was estimated from core depth and surface area of the sample. Dried samples were then ground using an electric spice grinder. Soil samples were then milled to fine powder in a mortar and pestle, sieved through a 2 mm mesh, and packed into tin capsules [29]. All samples were acidified with 2% HCl and analyzed for total organic carbon. Branford samples were analyzed using an Eltra^®^ CS580 elemental analyzer (Eltra GmbH, Haan, Germany) at the Yale Analytical and Stable Isotope Center (YASIC) while Quinnipiac Meadows samples were analyzed at the University of California, Merced Stable Isotope Ecosystems Laboratory using a Costech 4010 Elemental Analyzer (Costech Analytical Technologies Inc., Valencia, CA, USA) coupled with a Delta V Plus Continuous Flow Isotope Ratio Mass Spectrometer (Thermo Fisher Scientific, Bremen, Germany). These yielded carbon percentages in each sample, which were then used to estimate TOC. Data analysis was conducted in Microsoft Excel ^®^ and program R v.4.3.0 [30]. Total organic carbon (g ha^−1^) stock of each sample was estimated using the equation below [29] (Equation (1)).

(1) $${\mathrm{TOC}}_{\mathrm{stock}}=\%C\;\times \;\rho_{b}\;\times \;D\;\times \;100$$

where:

%C = Percent carbon in sample

ρ_b_ = Soil dry bulk density (g cm^−3^)

D = Soil depth (cm)

A sub-sample of remaining dry soil samples underwent additional sorting to extract all traces of below-ground live and dead rhizomes and fine roots to determine the vegetation fraction within the soils. Below-ground vegetative material was rinsed, patted dry and dried at 60 °C for 24 h. The proportion of vegetative matter (roots) was then estimated as a fraction of the dry sub-sample weight. All response variables were tested for normality using a Shapiro–Wilk test [30].

## 3. Results

**Figure 2:**
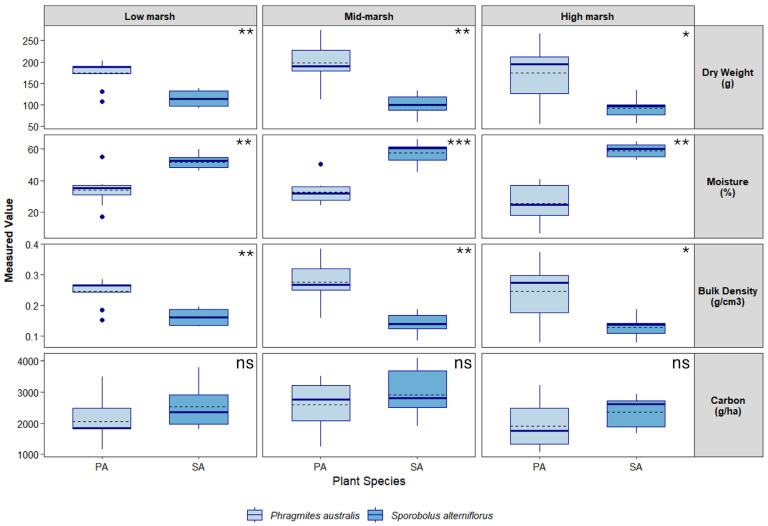
Soil parameters by zone collected between the fall of 2022 and winter of 2023 under two dominant species in a tidal salt marsh located in Branford, CT. Boxplots show median (solid line), mean (dashed line), interquartile range (box), range (whisker) and outliers. Asterisks at top right of each panel denote statistical significance between species (* = *p* < 0.05, ** = *p* < 0.001, *** = *p* < 0.0001, ns = not significant).

**Figure 3:**
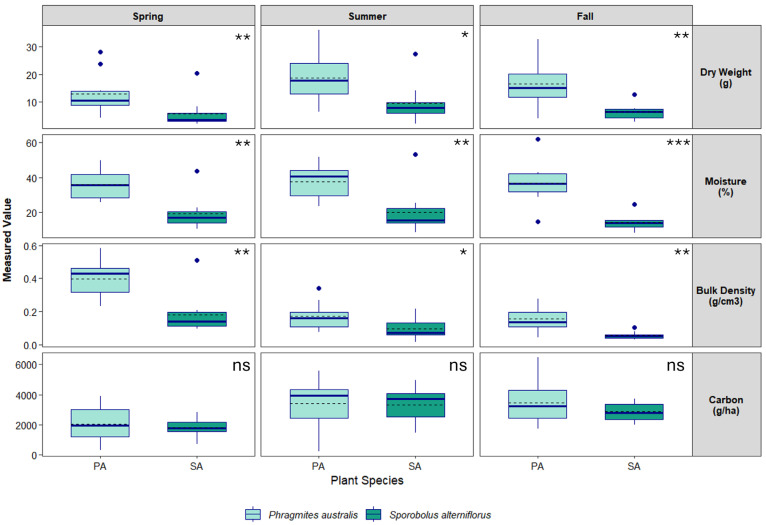
Seasonal variation in dry weight, percent moisture, bulk density and total organic carbon (TOC) content of soils collected over the spring, summer and fall of 2024 under two dominant species in a tidal salt marsh located in Quinnipiac Meadows, CT. Boxplots show median (solid line), mean (dashed line), interquartile range (box), range (whisker) and outliers. Asterisks at top right of each panel denote statistical significance between species (* = *p* < 0.05, ** = *p* < 0.001, *** = *p* < 0.0001, ns = not significant).

A total of 48 soil samples were collected over a 5-month period between October 2022 and February 2023 at Branford and 60 samples from Quinnipiac Meadows from April to November 2024 (Table S1/Supplementary Materials). Soils at Quinnipiac Meadows were drier than those at Branford, which resulted in shallower samples. Below-ground vegetative matter, including dead and live roots and rhizomes, constituted 10–11% of soil samples under both species. A Shapiro–Wilk test run on soil data from both sites revealed that bulk density data was significantly non-normal (Branford *W* = 0.95, *p* < 0.01; Quinnipiac Meadows *W* = 0.86, *p* < 0.001), as was dry weight (Branford *W* = 0.97, *p* < 0.01; Quinnipiac Meadows W = 0.88, *p* < 0.001). Consequently, a non-parametric Wilcoxon rank sum test was used to compare between species. Results indicated a significant difference in bulk density and dry weight between species at both locations, with *P. australis* generally exhibiting higher variability and higher mean and median bulk density values compared to *S. alterniflorus* (Table 1 and Table 2). Soil moisture content was greater under *S. alterniflorus* at Branford but greater in *P. australis* at Quinnipiac Meadows (Figure 2 and Figure 3).

Total organic carbon (TOC; kg C ha^−1^) varied spatially and temporally but was greater in *S. alterniflorus* than *P. australis* at Branford and vice versa for Quinnipiac Meadows (Figure 2 and Figure 3). A Scheirer–Ray–Hare test revealed no interaction between species and zone at Branford (*p* = 0.89), and Welch’s ANOVA detected a significant effect of season (*F* = 12.52, *p* < 0.001) but no effect by species at Quinnipiac Meadows. The post-hoc Dunn’s Test found significantly greater TOC in *S. alterniflorus* across all zones (*p* = 0.04).

## 4. Discussion

### 4.1. Alterations in Soil Physical Architecture and Geomorphology

The results of this study highlight a distinct and functional divergence in the physical soil properties between the invasive *Phragmites australis* and the native *Sporobolus alterniflorus*, even in the absence of significant disparities in overall carbon storage capacities. While general plant characteristics—such as above-ground biomass, stand density, and height—followed expected phenological patterns [31], the subsurface interactions reveal a more complex narrative. Our bulk density estimates align with regional observations from St. Lawrence Bay [15], though they fall below values recorded in southern ranges [31]. However, the critical finding lies in the significantly higher and more variable bulk density associated with *P. australis*.

This variance suggests that *P. australis* acts as a distinct geomorphic engineer compared to its native counterpart. The elevated bulk density indicates that *P. australis* likely facilitates higher rates of mineral sediment deposition or possesses a root architecture robust enough to physically compact the surrounding substrate to a greater degree than *S. alterniflorus*. This aligns with established research indicating that *P. australis* increases surface elevation in tidal marshes more rapidly than native species, utilizing a dual mechanism of organic matter accumulation and the efficient trapping of inorganic sediments [10]. The pronounced variance within the *P. australis* data further supports the “plasticity” hypothesis; this species is renowned for its ability to colonize a wider spectrum of micro-topographies and salinity gradients. Consequently, *P. australis* creates a more heterogeneous soil environment, contrasting sharply with the relatively uniform, monocultural stands typical of *S. alterniflorus* [32].

Above-ground vegetation, which influences soil carbon accumulation through organic matter inputs and root production, may respond strongly to interannual climate variability. Although *P. australis* is highly adaptable and occurs across a broad climatic range from temperate to tropical regions [33,34], our anecdotal observations at Branford suggest that the proximity of most *P. australis* stands to a regularly mowed walking path, coupled with the higher elevation and drier conditions, may have contributed to lower soil carbon in 2023. By contrast, *S. alterniflorus* occurred at lower elevations, closer to the water’s edge, thereby experiencing more frequent flooding and limiting carbon decomposition. Furthermore, the growing season of 2022 was marked by severe drought which may have limited all vegetation growth and carbon deposition [23]. *Sporobolus alterniflorus* typically performs best under moderate temperatures and precipitation but is sensitive to environmental extremes such as prolonged drought or excessive flooding [35]. In 2024, hydrologic and thermal extremes such as excessive flooding and subsequent inundation may have imposed physiological stress on *S. alterniflorus*, limiting growth and reducing biomass.

### 4.2. Carbon Storage Dynamics: Quantity vs. Density

The transition in salt marsh vegetation composition from native to invasive does not equate to a collapse in carbon sequestration potential, challenging the assumption that native marshes are invariably superior carbon sinks. The lack of a significant difference in total carbon stock between the two species contradicts literature suggesting that *P. australis* significantly outperforms native marshes [36], while also complicating views that native species are superior due to litter quality [37].

Instead, our data suggests a compensatory mechanism: while *P. australis* plots may exhibit lower carbon percentages per unit of soil, the total mass of carbon stored remains competitively high because the soil is more densely packed. This density-dependent storage suggests that *P. australis* creates a physically heavier carbon platform. There is a discrepancy between our findings and studies favoring native storage [37] hydrology, sediment supply, and microbial activity as significant drivers of carbon stocks. For instance, while *S. alterniflorus* may produce recalcitrant litter that stabilizes soil carbon, *P. australis* may encourage higher mineralization rates by promoting microbial decomposition [38]. Additionally, the variability in our *P. australis* carbon data may reflect its flexibility in below-ground biomass allocation, a trait responsive to nutrient availability and flooding stress [16]. The inconsistency among various studies strengthens the argument that local environmental drivers often override species-specific carbon traits.

### 4.3. Nutrient Synergy and Structural Integrity

When viewed from the perspective of nutrient enrichment, high nutrient runoff along coastal habitats of the Long Island Sound may stimulate above-ground growth at the expense of below-ground root biomass. In *S. alterniflorus* marshes, this allocational shift can be catastrophic, leading to “marsh collapse” where the root mat weakens, causing the soil to disintegrate [4]. On the other hand, *P. australis*, a documented “nitriphile” is capable of rapidly absorbing excess nutrients to fuel massive biomass production without suffering the same level of below-ground structural degradation [39]. Its high nitrogen demand drives it to extract deep nitrogen stores, a trait that fuels its invasiveness [18] but also results in a denser soil matrix. Therefore, the higher bulk density and variable carbon distribution in *P. australis* stands suggest this species may be more effective at “locking away” nutrients into a dense soil matrix. In eutrophic coastal waters, *P. australis* may function as a superior bio-filter compared to *S. alterniflorus*, stabilizing the physical platform even as it alters the biological community.

### 4.4. Resilience to Sea-Level Rise: The “Islands of Resilience”

The observed heterogeneity in *P. australis* soil properties may have profound implications for resilience against sea-level rise (SLR). The high variance in carbon stocks and bulk density suggests a trade-off: while *P. australis* builds a denser physical platform, its sequestration is less spatially uniform. However, in the context of rapid SLR, this heterogeneity may be advantageous. The “hot spots” of high carbon and high bulk density within *P. australis* stands may function as “islands of resilience.” These dense patches could resist drowning and erosion more effectively than the uniform, but potentially slower-accreting, *S. alterniflorus* stands [40]. Since higher bulk density typically correlates with increased soil shear strength [41], the invasion of *P. australis* may represent a shift toward a physically “hardened” coastline, capable of withstanding higher wave energy and deeper inundation, albeit at the cost of the micro-topographical uniformity preferred by some native fauna.

Despite the suggested geomorphic advantages of the establishment of non-native *P. australis*, it comes with significant ecological trade-offs. The tendency of *P. australis* to form dense, near-monocultural stands simplifies habitat structure, potentially reducing biodiversity and altering trophic pathways for native avian and macroinvertebrate communities [32,42]. Thus, the invasion represents a transition from the biological heterogeneity and specialized habitat niches of *S. alterniflorus* to a more physically stable but biologically uniform state.

## 5. Conclusions

We found no consistent evidence to suggest that the invasion of Connecticut’s salt marshes by non-native plants significantly impacts carbon stocks. However, given the known losses in wildlife habitat and diversity, we propose that the transition from *S. alterniflorus* to *P. australis* represents a trade-off in ecological functions. Further exploration of the role played by hydrological regimes, sediment supply, and nutrient loading characteristics across multiple salt marshes of the Long Island Sound would clarify the extent to which the observed patterns are locally driven versus broadly generalized.

The implications of this study extend into the management of coastal wetlands in the Anthropocene. As sea levels continue to rise and anthropogenic nutrient loading persists, the restoration preference for *S. alterniflorus* must be weighed against the geomorphic stability provided by *P. australis*. While traditionally managed as an aggressive invasive threat to biodiversity, *P. australis* demonstrates a capacity to maintain high bulk density and substantial carbon stocks under conditions that might destabilize native marshes.

Consequently, in areas subject to rapid subsidence, high wave energy, or extreme eutrophication, *P. australis* may provide a critical “geomorphic service.” The species’ ability to create a dense, heterogeneous, and nutrient-absorbent soil matrix suggests it could act as a buffer against marsh collapse. Future management strategies should consider this functional trade-off: the preservation of native biodiversity versus the maintenance of a physically resilient marsh platform capable of keeping pace with rising seas. The “islands of resilience” created by *P. australis* may well prove to be essential components of coastal defense in a rapidly changing climate.

## Figures and Tables

**Table 1:** Mean soil parameters along transects surveyed at a tidal salt marsh in Branford, CT from October 2022 to February 2023. Mean elevation at the high marsh was approximately 0.7 m above sea level.

	Species	*N*	High Marsh $(\bar{x}\;\pm$ SE)	Mid Marsh $(\bar{x}\;\pm$ SE)	Low Marsh $(\bar{x}\;\pm$ SE)
Dry weight (g)	*P. australis*	9	175.33 ± 22.67	197.25 ± 17.39	174.49 ± 10.87
	*S. alterniflorus*	7	91.98 ± 9.31	102.70 ± 8.12	116.02 ± 6.21
Bulk density (g cm^−3^)	*P. australis*	9	0.26 ± 0.03	0.28 ± 0.02	0.24 ± 0.02
	*S. alterniflorus*	7	0.14 ± 0.03	0.14 ± 0.01	0.16 ± 0.01
Carbon weight percent (%)	*P. australis*	9	30.00 ± 4.13	31.91 ± 2.89	29.67 ± 5.77
	*S. alterniflorus*	7	56.04 ± 8.24	74.90 ± 9.68	52.00 ± 4.21
Total Organic Carbon (g ha^−1^)	*P. australis*	9	1915.40 ± 239.23	2596.41 ± 267.58	2065.79 ± 249.58
	*S. alterniflorus*	7	2351.09 ± 197.21	3065.70 ± 266.47	2522.15 ± 217.15

**Table 2:** Seasonal variations in soil parameters along transects surveyed at Quinnipiac Meadows over the spring, summer and fall of 2024.

	Species	*N*	Spring $(\bar{x}\;\pm$ SE)	Summer $(\bar{x}\;\pm$ SE)	Fall $(\bar{x}\;\pm$ SE)
Dry weight (g)	*P. australis*	10	12.92 ± 8.37	18.63 ± 2.82	16.46 ± 2.61
	*S. alterniflorus*	10	5.78 ± 1.74	9.52 ± 2.33	6.34 ± 0.88
Bulk density (g cm^−3^)	*P. australis*	10	0.40 ± 0.03	0.17 ± 0.03	0.16 ± 0.02
	*S. alterniflorus*	10	0.18 ± 0.04	0.10 ± 0.02	0.06 ± 0.01
Carbon weight percent (%)	*P. australis*	10	8.13 ± 1.34	9.45 ± 1.67	11.06 ± 2.09
	*S. alterniflorus*	10	19.03 ± 2.66	20.84 ± 2.68	22.30 ± 1.87
Total Organic Carbon (g ha^−1^)	*P. australis*	10	2035.10 ± 367.45	3389.83 ± 528.19	3438.59 ± 480.02
	*S. alterniflorus*	10	1794.80 ± 185.53	3324.65 ± 400.73	2849.43 ± 200.84

## Data Availability

The raw data supporting the conclusions of this article will be made available by the authors on request.

## Supplementary Materials

The following supporting information can be downloaded at: https://www.mdpi.com/article/10.3390/biology15040315/s1, Table S1: 60 samples from Quinnipiac Meadows from April to November 2024.
